# Crystal structure of the di-Mannich base 4,4′-di­chloro-3,3′,5,5′-tetra­methyl-2,2′-[imidazolidine-1,3-diylbis(methyl­ene)]diphenol

**DOI:** 10.1107/S2056989015002212

**Published:** 2015-02-25

**Authors:** Augusto Rivera, Luz Stella Nerio, Michael Bolte

**Affiliations:** a Departamento de Química, Facultad de Ciencias, Universidad Nacional de Colombia, Sede Bogotá, Cra 30 No. 45-03, Bogotá, Colombia; b Institut für Anorganische Chemie, Goethe-Universität, Max-von-Laue-Strasse 7, Frankfurt/Main D-60438, Germany

**Keywords:** crystal structure, imidazolidine, di-Mannich base, hydrogen bonding, *syn* conformation

## Abstract

The title compound, C_21_H_26_Cl_2_N_2_O_2_, was prepared in a solvent-free microwave-assisted synthesis, and crystallizes in the ortho­rhom­bic space group *Pna*2_1_. The imidazolidine ring adopts an envelope conformation and its mean plane is almost perpendicular to the two pendant aromatic rings [dihedral angles = 84.61 (9) and 86.54 (9)°]. The mol­ecular structure shows the presence of two intra­molecular O—H⋯N hydrogen bonds between the phenolic hy­droxy groups and imidazolidine N atoms. The two 3-chloro-6-hy­droxy-2,4-di­methyl­benzyl groups are located in a *cis* orientation with respect to the imidazolidine fragment. As a result, the lone pairs of electrons on the N atoms are presumed to be disposed in a *syn* conformation. This is therefore the first example of an exception to the ‘rabbit-ears’ effect in such 2,2′-[imidazolidine-1,3-diylbis(methyl­ene)]diphenol derivatives.

## Abstract 2

In the title compound, the imidizadoline ring adopts an envelope conformation and the nitro­gen lone pairs are oriented in a *syn* disposition. The crystal packing is stabilized by C—H⋯O hydrogen-bonding inter­actions.

## Chemical context   

As a continuation of our investigations of the Mannich reaction, we have synthesized a family of compounds of the type 2,2′-[imidazolidine-1,3-diylbis(methyl­ene)]di(hydroxyar­yl), from reactions between 1,3,6,8-tetra­zatri­cyclo­[4.4.1.1^3,8^]dodecane (TATD) and phenols or naphthols (Rivera *et al.*, 1993 ▸, 2005 ▸; Rivera & Quevedo, 2013 ▸). Such compounds are known to be valuable in homogeneous catalysis (Kober *et al.*, 2012 ▸) and for the preparation of tetra­hydro­salens (Rivera *et al.*, 2004 ▸) and heterocalixarenes (Rivera & Quevedo, 2004 ▸). Mannich bases are also convenient models for studying the nature of hydrogen bonding and other weak non-covalent inter­actions, as they contain at least one phenolic or naphtho­lic hy­droxy group as a proton donor, as well as an *ortho*-amino­methyl­group as a proton acceptor in the same mol­ecule (Koll *et al.*, 2006 ▸). Herein, as part of our systematic investigations of di-Mannich bases as convenient model systems for the study of intra­molecular proton-transfer processes, we report the mol­ecular and crystal structure of the title di-Mannich base, 4,4′-di­chloro-3,3′,5,5′-tetra­methyl-2,2′- [imidazolidine-1,3-diylbis(methyl­ene)]diphenol (I).
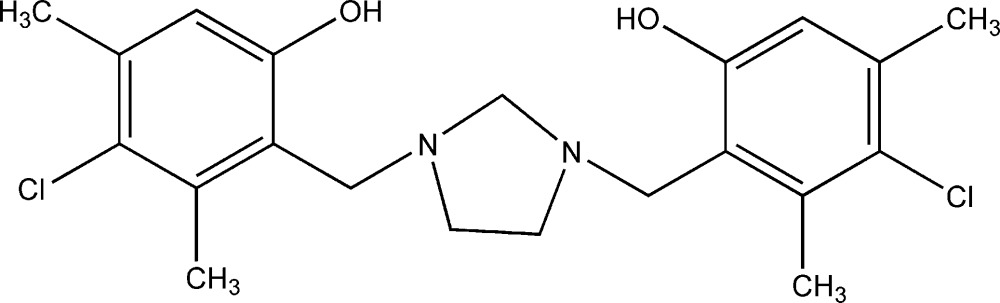


In a previous report (Rivera & Quevedo, 2013 ▸), the title compound (I) was obtained under solvent-free conditions by heating a 1:4 mixture of TATD and 4-chloro-3,5-di­methyl­phenol in an oil bath with stirring at 423 K for 20 min. Drawbacks of this synthesis include the long reaction time and a requirement of considerable effort to optimize the reaction conditions and temperature control. We therefore subsequently explored this reaction under solvent-free, microwave-assisted conditions. The reaction was found to proceed smoothly under microwave irradiation in only 3 min at 403 K, in modest yield.

## Structural commentary   

**Figure 1:**
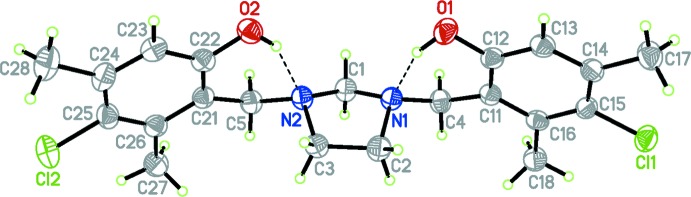
The title mol­ecule, showing the atom-numbering scheme. Displacement ellipsoids are drawn at the 50% probability level. Hydrogen bonds are shown as dashed lines.

In the title mol­ecule (I), Fig. 1 ▸, the imidazolidine ring adopts an envelope conformation, with atom C1 at the flap. The mol­ecular structure shows two intra­molecular O—H⋯N hydrogen bonds (Table 1 ▸) with *S*(6) graph-set motifs between the hy­droxy groups of the substituted phenol rings and the two imidazolidine N atoms. The benzyl groups are located in an unexpected 1,3-diequatorial *syn* arrangement on the heterocyclic ring with dihedral angles between the mean plane through the N1/C2/C3/N2 atoms of the imidazolidine ring and the C11–C16 and C21–C26 aromatic rings of 84.61 (9) and 88.54 (9)°, respectively. The non-bonding electron pairs on the imidazolidine N atoms that are involved in both intra- and inter­molecular hydrogen-bonding inter­actions adopt an unusual *syn* arrangement. As such, this mol­ecule defies the well known ‘rabbit-ears’ effect (Hutchins *et al.*, 1968 ▸) in which N–CH_2_–N systems adopt *anti* conformations to avoid repulsions between the nitro­gen lone pairs. Although in the very similar structure of *meso*-4,4′-di­fluoro-2,2′-{[(3a*R*,7a*S*)-2,3,3a,4,5,6,7,7a-octa­hydro-1*H*-1,3-benzimidazole-1,3-di­yl]bis(methyl­ene)}diphenol (Rivera *et al.*, 2013 ▸) the N-atom lone pairs are *syn*, mol­ecule (I) is the first reported exception to the ‘rabbit-ears’ effect in compounds of the 2,2′-[imidazolidine-1,3-diylbis(methyl­ene)]diphenol type (Rivera *et al.*, 2011 ▸, 2012*a* ▸,*b* ▸,*c* ▸, 2013 ▸, 2014 ▸).

## Supra­molecular features   

**Figure 2:**
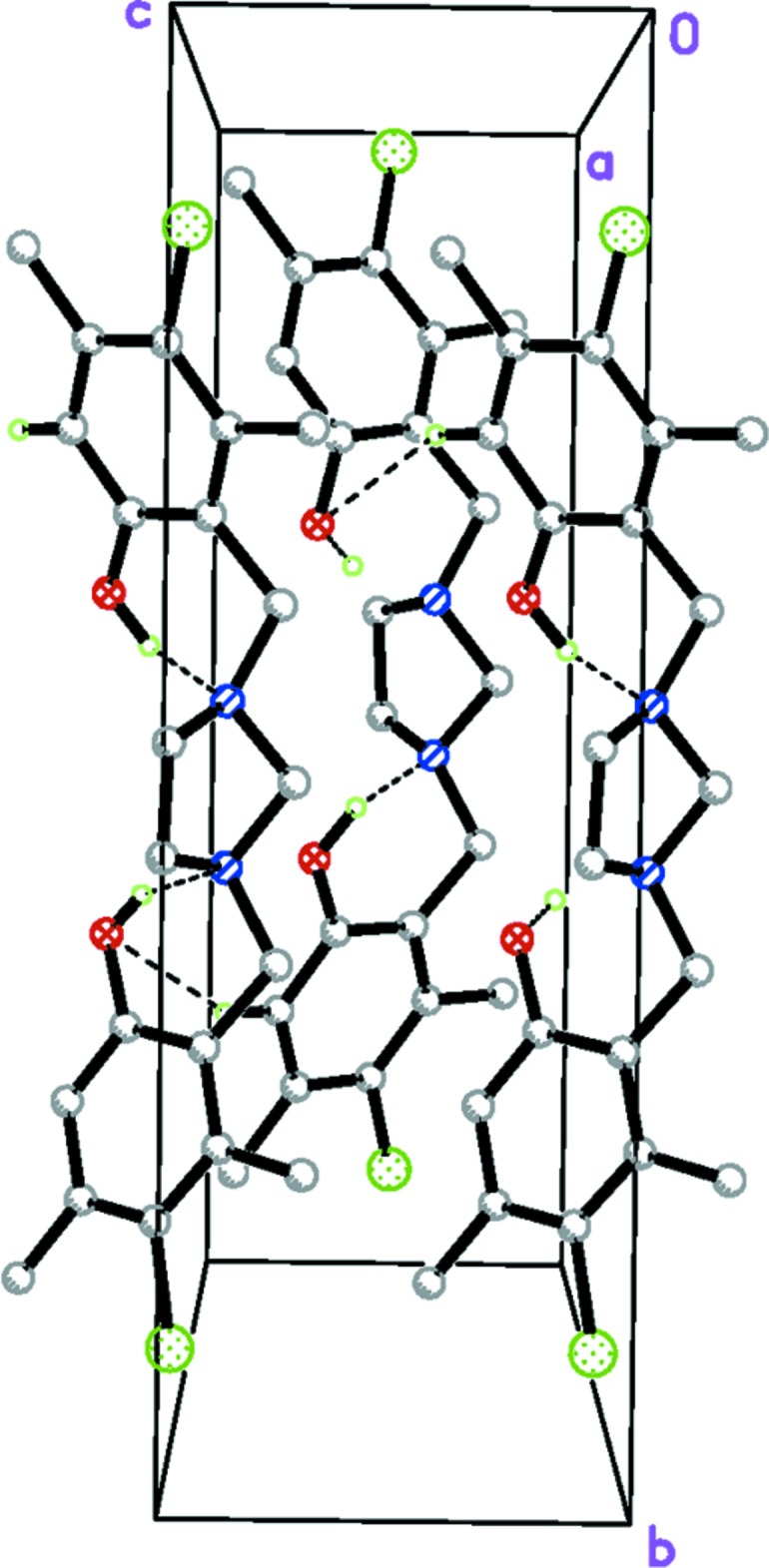
A perspective view along the *a* axis of the crystal packing of the title compound,. The C—H⋯O hydrogen bonds are shown as dashed lines.

With both hy­droxy groups of (I) involved in intra­molecular hydrogen bonds, the only directional interaction in the crystal is a C13—H13⋯O2^i^ bond (Table 1 ▸ and Fig. 2 ▸), which links adjacent mol­ecules in a head-to-tail fashion into zigzag chains, extending along the *c-*axis direction (Fig. 2 ▸).

## Database survey   

A search in the Cambridge Structural Database (Groom & Allen 2014 ▸) revealed previous reports of six structures of related 2,2′-[imidazolidine-1,3-diylbis(methyl­ene)]diphenol compounds (Rivera *et al.*, 2011 ▸, 2012*a* ▸,*b* ▸,*c* ▸, 2013 ▸, 2014 ▸). Each of these also shows intra­molecular O—H⋯N hydrogen bonds between the two imidazolidine N atoms and the hy­droxy groups. In addition, the *D*⋯*A* distances in these compounds compare well with those observed in the title compound. As with (I), the imidazolidine ring in the *p-tert-*butyl­phenol derivative (Rivera *et al.*, 2013 ▸), adopts an envelope conformation whereas, in the other five the ring adopts a twist conformation. Furthermore, unlike the title compound, the nitro­gen lone pairs in all six of the related derivatives are oriented in an *anti* disposition.

## Synthesis and crystallization   

A mixture of 1,3,6,8-tetra­zatri­cyclo­[4.4.1.1^3,8^]dodecane (0.100 g, 0.6 mmol) and 4-chloro-3,5-di­methyl­phenol (0.375 g, 2.4 mmol) without any solvent was exposed to microwave irradiation in a CEM Discover reactor (with 250 W as the maximum power) for 3 min at a temperature of 403 K. Once cooled to room temperature, the reaction mixture was dissolved with CHCl_3_ which was removed under reduced pressure to yield the crude product. This was further purified by column chromatography on silica gel using a mixture of benzene:ethyl acetate (80:20) as eluent (yield 21%, m.p. = 421–422 K). Single crystals in the form of needles shorter than 1 mm were obtained from a chloro­form:ethanol (50:50) solution by slow evaporation of the solvent at room temperature over a period of one week.

## Refinement   

Crystal data, data collection and structure refinement details are summarized in Table 2 ▸. All the H atoms were located in difference electron density maps. The hy­droxy H atoms were freely refined. C-bound H atoms were fixed geometrically (C—H = 0.95 to 0.99 Å) and refined using a riding model, with *U*_iso_(H) set to 1.2*U*_eq_ (1.5*U*_eq_ for methyl groups) of the parent atoms. The methyl groups were allowed to rotate but not to tip.

## Supplementary Material

Crystal structure: contains datablock(s) I, New_Global_Publ_Block. DOI: 10.1107/S2056989015002212/sj5442sup1.cif

Structure factors: contains datablock(s) I. DOI: 10.1107/S2056989015002212/sj5442Isup2.hkl

Supporting information file. DOI: 10.1107/S2056989015002212/sj5442Isup3.cml

CCDC reference: 1046907

Additional supporting information:  crystallographic information; 3D view; checkCIF report

## Figures and Tables

**Table 1:** Hydrogen-bond geometry (, )

*D*H*A*	*D*H	H*A*	*D* *A*	*D*H*A*
O1H1N1	0.99(5)	1.66(5)	2.606(3)	158(4)
O2H2N2	0.86(4)	1.83(4)	2.619(3)	152(3)
C13H13O2^i^	0.95	2.59	3.464(4)	152

Symmetry code: (i) 
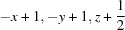
.

**Table 2:** Experimental details

Crystal data	
Chemical formula	C_21_H_26_Cl_2_N_2_O_2_
*M*_r_	409.34
Crystal system, space group	Orthorhombic, *P* *n* *a*2_1_
Temperature (K)	173
*a*, *b*, *c* ()	20.1594(11), 17.8088(12), 5.6120(3)
*V* (^3^)	2014.8(2)
*Z*	4
Radiation type	Mo *K*
(mm^1^)	0.34
Crystal size (mm)	0.22 0.11 0.09

Data collection	
Diffractometer	Stoe *IPDS* II two circle
Absorption correction	Multi-scan (*X-AREA*; Stoe Cie, 2001 ▸)
*T*_min_, *T*_max_	0.891, 0.946
No. of measured, independent and observed [*I* > 2(*I*)] reflections	17730, 3708, 3280
*R*_int_	0.080
(sin /)_max_ (^1^)	0.604

Refinement	
*R*[*F* ^2^ > 2(*F* ^2^)], *wR*(*F* ^2^), *S*	0.035, 0.082, 1.00
No. of reflections	3708
No. of parameters	256
No. of restraints	1
H-atom treatment	H atoms treated by a mixture of independent and constrained refinement
_max_, _min_ (e ^3^)	0.16, 0.20
Absolute structure	Flack *x* determined using 1338 quotients [(*I* ^+^)(*I* )]/[(*I* ^+^)+(*I* )] (Parsons *et al.*, 2013 ▸)
Absolute structure parameter	0.00(4)

Computer programs: *X-AREA* and *X-RED32* (Stoe Cie, 2001 ▸), *SHELXS87* and *XP* in *SHELXTL-Plus* (Sheldrick, 2008 ▸), *SHELXL2014* (Sheldrick, 2015 ▸).

## supplementary crystallographic information

### Crystal data

**Table:**

C_21_H_26_Cl_2_N_2_O_2_	*D*_x_ = 1.349 Mg m^−^^3^
*M**_r_* = 409.34	Mo *K*α radiation, λ = 0.71073 Å
Orthorhombic, *P**n**a*2_1_	Cell parameters from 16491 reflections
*a* = 20.1594 (11) Å	θ = 2.1–25.9°
*b* = 17.8088 (12) Å	µ = 0.34 mm^−^^1^
*c* = 5.6120 (3) Å	*T* = 173 K
*V* = 2014.8 (2) Å^3^	Needle, colourless
*Z* = 4	0.22 × 0.11 × 0.09 mm
*F*(000) = 864	

### Data collection

**Table:**

Stoe IPDS II two-circle diffractometer	3280 reflections with *I* > 2σ(*I*)
Radiation source: Genix 3D IµS microfocus X-ray source	*R*_int_ = 0.080
ω scans	θ_max_ = 25.4°, θ_min_ = 2.0°
Absorption correction: multi-scan (*X-AREA*; Stoe & Cie, 2001)	*h* = −24→24
*T*_min_ = 0.891, *T*_max_ = 0.946	*k* = −21→21
17730 measured reflections	*l* = −6→6
3708 independent reflections	

### Refinement

**Table:**

Refinement on *F*^2^	Hydrogen site location: mixed
Least-squares matrix: full	H atoms treated by a mixture of independent and constrained refinement
*R*[*F*^2^ > 2σ(*F*^2^)] = 0.035	*w* = 1/[σ^2^(*F*_o_^2^) + (0.0492*P*)^2^] where *P* = (*F*_o_^2^ + 2*F*_c_^2^)/3
*wR*(*F*^2^) = 0.082	(Δ/σ)_max_ = 0.001
*S* = 1.00	Δρ_max_ = 0.16 e Å^−^^3^
3708 reflections	Δρ_min_ = −0.20 e Å^−^^3^
256 parameters	Absolute structure: Flack *x* determined using 1338 quotients [(*I*^+^)-(*I*^-^)]/[(*I*^+^)+(*I*^-^)] (Parsons *et al.*, 2013)
1 restraint	Absolute structure parameter: 0.00 (4)

### Special details

**Table:**

Geometry. All e.s.d.'s (except the e.s.d. in the dihedral angle between two l.s. planes) are estimated using the full covariance matrix. The cell e.s.d.'s are taken into account individually in the estimation of e.s.d.'s in distances, angles and torsion angles; correlations between e.s.d.'s in cell parameters are only used when they are defined by crystal symmetry. An approximate (isotropic) treatment of cell e.s.d.'s is used for estimating e.s.d.'s involving l.s. planes.

### Fractional atomic coordinates and isotropic or equivalent isotropic displacement parameters (Å^2^)

**Table:**

	*x*	*y*	*z*	*U*_iso_*/*U*_eq_	
Cl1	0.71322 (3)	0.87262 (4)	0.49746 (17)	0.04042 (19)	
Cl2	0.63887 (4)	0.04441 (4)	0.5199 (2)	0.0535 (2)	
O1	0.55382 (11)	0.60445 (11)	0.6973 (4)	0.0383 (5)	
H1	0.574 (2)	0.565 (3)	0.595 (9)	0.074 (13)*	
O2	0.52557 (10)	0.34212 (11)	0.6988 (4)	0.0368 (5)	
H2	0.5460 (19)	0.3744 (19)	0.611 (7)	0.044 (10)*	
N1	0.62589 (11)	0.52659 (13)	0.4013 (5)	0.0304 (5)	
N2	0.61125 (12)	0.40126 (13)	0.4025 (5)	0.0308 (5)	
C1	0.60746 (16)	0.46539 (14)	0.2433 (5)	0.0326 (6)	
H1A	0.5620	0.4721	0.1797	0.039*	
H1B	0.6390	0.4605	0.1090	0.039*	
C2	0.68434 (15)	0.49812 (15)	0.5313 (7)	0.0394 (7)	
H2A	0.6872	0.5204	0.6925	0.047*	
H2B	0.7258	0.5092	0.4435	0.047*	
C3	0.67177 (14)	0.41297 (15)	0.5448 (6)	0.0341 (7)	
H3A	0.7096	0.3846	0.4768	0.041*	
H3B	0.6649	0.3968	0.7118	0.041*	
C4	0.63685 (15)	0.59859 (15)	0.2782 (6)	0.0337 (6)	
H4A	0.6020	0.6056	0.1558	0.040*	
H4B	0.6803	0.5971	0.1959	0.040*	
C5	0.60838 (15)	0.32829 (16)	0.2813 (6)	0.0337 (6)	
H5A	0.6508	0.3195	0.1969	0.040*	
H5B	0.5725	0.3294	0.1608	0.040*	
C11	0.63568 (13)	0.66441 (15)	0.4475 (5)	0.0290 (6)	
C12	0.59219 (13)	0.66519 (15)	0.6419 (6)	0.0304 (6)	
C13	0.58553 (14)	0.72820 (15)	0.7842 (6)	0.0332 (6)	
H13	0.5558	0.7269	0.9154	0.040*	
C14	0.62147 (14)	0.79335 (15)	0.7393 (6)	0.0326 (7)	
C15	0.66605 (13)	0.79115 (14)	0.5501 (6)	0.0308 (6)	
C16	0.67502 (13)	0.72850 (15)	0.4053 (5)	0.0296 (6)	
C17	0.61244 (17)	0.86156 (17)	0.8953 (7)	0.0429 (8)	
H17A	0.5790	0.8509	1.0175	0.064*	
H17B	0.5977	0.9040	0.7976	0.064*	
H17C	0.6547	0.8741	0.9720	0.064*	
C18	0.72556 (15)	0.72805 (16)	0.2061 (6)	0.0387 (7)	
H18A	0.7530	0.7733	0.2163	0.058*	
H18B	0.7026	0.7270	0.0522	0.058*	
H18C	0.7538	0.6835	0.2208	0.058*	
C21	0.59596 (13)	0.26437 (14)	0.4523 (5)	0.0299 (6)	
C22	0.55305 (14)	0.27410 (15)	0.6465 (6)	0.0317 (6)	
C23	0.53458 (15)	0.21404 (16)	0.7892 (6)	0.0351 (6)	
H23	0.5045	0.2221	0.9169	0.042*	
C24	0.55924 (16)	0.14239 (16)	0.7492 (6)	0.0385 (7)	
C25	0.60474 (15)	0.13394 (15)	0.5636 (6)	0.0359 (7)	
C26	0.62373 (14)	0.19245 (16)	0.4129 (6)	0.0334 (7)	
C27	0.67213 (16)	0.18022 (16)	0.2127 (6)	0.0405 (7)	
H27A	0.6872	0.1279	0.2141	0.061*	
H27B	0.7103	0.2137	0.2333	0.061*	
H27C	0.6504	0.1911	0.0604	0.061*	
C28	0.5373 (2)	0.07780 (19)	0.9026 (7)	0.0534 (9)	
H28A	0.5161	0.0395	0.8029	0.080*	
H28B	0.5056	0.0958	1.0220	0.080*	
H28C	0.5759	0.0560	0.9829	0.080*	

### Atomic displacement parameters (Å^2^)

**Table:**

	*U*^11^	*U*^22^	*U*^33^	*U*^12^	*U*^13^	*U*^23^
Cl1	0.0421 (4)	0.0336 (3)	0.0456 (4)	−0.0080 (3)	0.0053 (4)	−0.0003 (4)
Cl2	0.0674 (5)	0.0306 (3)	0.0625 (6)	0.0073 (3)	−0.0007 (6)	0.0026 (4)
O1	0.0401 (11)	0.0349 (10)	0.0400 (14)	−0.0082 (9)	0.0115 (10)	0.0021 (9)
O2	0.0379 (11)	0.0373 (11)	0.0353 (13)	0.0040 (9)	0.0028 (10)	0.0014 (10)
N1	0.0329 (12)	0.0293 (11)	0.0289 (13)	−0.0008 (9)	−0.0042 (11)	0.0015 (10)
N2	0.0352 (13)	0.0284 (11)	0.0287 (13)	0.0008 (9)	−0.0056 (11)	0.0000 (10)
C1	0.0389 (15)	0.0314 (14)	0.0276 (17)	−0.0011 (11)	−0.0055 (13)	0.0012 (12)
C2	0.0415 (15)	0.0360 (14)	0.041 (2)	−0.0018 (12)	−0.0137 (17)	0.0032 (15)
C3	0.0362 (15)	0.0345 (13)	0.0316 (18)	0.0020 (11)	−0.0093 (13)	−0.0026 (13)
C4	0.0397 (16)	0.0294 (14)	0.0319 (17)	−0.0007 (11)	0.0015 (14)	0.0043 (12)
C5	0.0385 (15)	0.0328 (14)	0.0297 (17)	−0.0006 (12)	−0.0014 (13)	−0.0034 (13)
C11	0.0294 (14)	0.0299 (13)	0.0278 (18)	0.0023 (10)	−0.0001 (12)	0.0028 (11)
C12	0.0277 (14)	0.0317 (13)	0.0316 (17)	0.0003 (11)	0.0017 (12)	0.0036 (12)
C13	0.0314 (14)	0.0368 (14)	0.0314 (17)	0.0021 (12)	0.0052 (13)	0.0011 (12)
C14	0.0320 (14)	0.0318 (14)	0.0342 (19)	0.0028 (10)	−0.0001 (13)	−0.0008 (13)
C15	0.0286 (13)	0.0298 (13)	0.0341 (18)	−0.0021 (10)	−0.0026 (12)	0.0020 (12)
C16	0.0268 (13)	0.0325 (14)	0.0294 (16)	0.0033 (11)	0.0004 (12)	0.0048 (11)
C17	0.0484 (18)	0.0375 (16)	0.043 (2)	0.0007 (13)	0.0080 (16)	−0.0055 (14)
C18	0.0394 (16)	0.0375 (15)	0.0392 (19)	−0.0023 (12)	0.0099 (15)	−0.0007 (13)
C21	0.0295 (13)	0.0308 (13)	0.0294 (18)	−0.0022 (11)	−0.0035 (12)	−0.0018 (11)
C22	0.0309 (14)	0.0345 (14)	0.0297 (17)	0.0005 (11)	−0.0053 (12)	−0.0020 (12)
C23	0.0337 (15)	0.0418 (16)	0.0297 (16)	−0.0032 (12)	0.0003 (13)	0.0006 (13)
C24	0.0450 (17)	0.0352 (15)	0.0352 (19)	−0.0093 (12)	−0.0069 (15)	0.0043 (13)
C25	0.0403 (15)	0.0295 (13)	0.038 (2)	−0.0003 (11)	−0.0085 (13)	−0.0005 (12)
C26	0.0309 (14)	0.0346 (15)	0.0347 (17)	−0.0020 (11)	−0.0047 (13)	−0.0045 (12)
C27	0.0421 (17)	0.0390 (16)	0.040 (2)	0.0014 (13)	0.0049 (15)	−0.0065 (14)
C28	0.065 (2)	0.0424 (18)	0.053 (2)	−0.0127 (16)	0.0006 (19)	0.0101 (16)

### Geometric parameters (Å, º)

**Table:**

Cl1—C15	1.760 (3)	C13—C14	1.391 (4)
Cl2—C25	1.754 (3)	C13—H13	0.9500
O1—C12	1.366 (3)	C14—C15	1.392 (4)
O1—H1	0.99 (5)	C14—C17	1.508 (4)
O2—C22	1.364 (3)	C15—C16	1.392 (4)
O2—H2	0.86 (4)	C16—C18	1.513 (4)
N1—C1	1.453 (4)	C17—H17A	0.9800
N1—C4	1.473 (4)	C17—H17B	0.9800
N1—C2	1.476 (4)	C17—H17C	0.9800
N2—C1	1.452 (3)	C18—H18A	0.9800
N2—C5	1.468 (4)	C18—H18B	0.9800
N2—C3	1.473 (4)	C18—H18C	0.9800
C1—H1A	0.9900	C21—C22	1.402 (4)
C1—H1B	0.9900	C21—C26	1.415 (4)
C2—C3	1.539 (4)	C22—C23	1.387 (4)
C2—H2A	0.9900	C23—C24	1.388 (4)
C2—H2B	0.9900	C23—H23	0.9500
C3—H3A	0.9900	C24—C25	1.396 (5)
C3—H3B	0.9900	C24—C28	1.503 (4)
C4—C11	1.509 (4)	C25—C26	1.396 (4)
C4—H4A	0.9900	C26—C27	1.504 (5)
C4—H4B	0.9900	C27—H27A	0.9800
C5—C21	1.510 (4)	C27—H27B	0.9800
C5—H5A	0.9900	C27—H27C	0.9800
C5—H5B	0.9900	C28—H28A	0.9800
C11—C12	1.399 (4)	C28—H28B	0.9800
C11—C16	1.410 (4)	C28—H28C	0.9800
C12—C13	1.384 (4)		
			
C12—O1—H1	101 (3)	C15—C14—C17	122.9 (3)
C22—O2—H2	106 (2)	C14—C15—C16	123.5 (2)
C1—N1—C4	113.9 (2)	C14—C15—Cl1	117.0 (2)
C1—N1—C2	104.4 (2)	C16—C15—Cl1	119.5 (2)
C4—N1—C2	114.3 (2)	C15—C16—C11	118.5 (3)
C1—N2—C5	114.2 (2)	C15—C16—C18	121.5 (3)
C1—N2—C3	105.4 (2)	C11—C16—C18	119.9 (3)
C5—N2—C3	114.2 (2)	C14—C17—H17A	109.5
N2—C1—N1	101.6 (2)	C14—C17—H17B	109.5
N2—C1—H1A	111.5	H17A—C17—H17B	109.5
N1—C1—H1A	111.5	C14—C17—H17C	109.5
N2—C1—H1B	111.5	H17A—C17—H17C	109.5
N1—C1—H1B	111.5	H17B—C17—H17C	109.5
H1A—C1—H1B	109.3	C16—C18—H18A	109.5
N1—C2—C3	103.4 (2)	C16—C18—H18B	109.5
N1—C2—H2A	111.1	H18A—C18—H18B	109.5
C3—C2—H2A	111.1	C16—C18—H18C	109.5
N1—C2—H2B	111.1	H18A—C18—H18C	109.5
C3—C2—H2B	111.1	H18B—C18—H18C	109.5
H2A—C2—H2B	109.1	C22—C21—C26	118.5 (3)
N2—C3—C2	104.4 (2)	C22—C21—C5	120.2 (2)
N2—C3—H3A	110.9	C26—C21—C5	121.2 (3)
C2—C3—H3A	110.9	O2—C22—C23	116.8 (3)
N2—C3—H3B	110.9	O2—C22—C21	121.9 (3)
C2—C3—H3B	110.9	C23—C22—C21	121.3 (3)
H3A—C3—H3B	108.9	C22—C23—C24	121.3 (3)
N1—C4—C11	112.2 (3)	C22—C23—H23	119.4
N1—C4—H4A	109.2	C24—C23—H23	119.4
C11—C4—H4A	109.2	C23—C24—C25	117.1 (3)
N1—C4—H4B	109.2	C23—C24—C28	120.4 (3)
C11—C4—H4B	109.2	C25—C24—C28	122.6 (3)
H4A—C4—H4B	107.9	C26—C25—C24	123.5 (3)
N2—C5—C21	112.3 (2)	C26—C25—Cl2	119.1 (2)
N2—C5—H5A	109.1	C24—C25—Cl2	117.4 (2)
C21—C5—H5A	109.1	C25—C26—C21	118.2 (3)
N2—C5—H5B	109.1	C25—C26—C27	121.5 (3)
C21—C5—H5B	109.1	C21—C26—C27	120.3 (3)
H5A—C5—H5B	107.9	C26—C27—H27A	109.5
C12—C11—C16	118.4 (3)	C26—C27—H27B	109.5
C12—C11—C4	120.6 (2)	H27A—C27—H27B	109.5
C16—C11—C4	120.9 (3)	C26—C27—H27C	109.5
O1—C12—C13	117.1 (3)	H27A—C27—H27C	109.5
O1—C12—C11	121.6 (3)	H27B—C27—H27C	109.5
C13—C12—C11	121.2 (3)	C24—C28—H28A	109.5
C12—C13—C14	121.4 (3)	C24—C28—H28B	109.5
C12—C13—H13	119.3	H28A—C28—H28B	109.5
C14—C13—H13	119.3	C24—C28—H28C	109.5
C13—C14—C15	116.8 (3)	H28A—C28—H28C	109.5
C13—C14—C17	120.3 (3)	H28B—C28—H28C	109.5
			
C5—N2—C1—N1	−168.1 (2)	C14—C15—C16—C18	−178.2 (3)
C3—N2—C1—N1	−42.0 (3)	Cl1—C15—C16—C18	1.3 (4)
C4—N1—C1—N2	170.7 (2)	C12—C11—C16—C15	−3.7 (4)
C2—N1—C1—N2	45.4 (3)	C4—C11—C16—C15	172.2 (3)
C1—N1—C2—C3	−31.0 (3)	C12—C11—C16—C18	176.3 (3)
C4—N1—C2—C3	−156.0 (2)	C4—C11—C16—C18	−7.8 (4)
C1—N2—C3—C2	22.5 (3)	N2—C5—C21—C22	37.3 (4)
C5—N2—C3—C2	148.6 (3)	N2—C5—C21—C26	−146.7 (3)
N1—C2—C3—N2	5.2 (3)	C26—C21—C22—O2	178.5 (3)
C1—N1—C4—C11	163.0 (2)	C5—C21—C22—O2	−5.4 (4)
C2—N1—C4—C11	−77.2 (3)	C26—C21—C22—C23	−4.0 (4)
C1—N2—C5—C21	−166.3 (2)	C5—C21—C22—C23	172.1 (3)
C3—N2—C5—C21	72.3 (3)	O2—C22—C23—C24	179.3 (3)
N1—C4—C11—C12	−36.2 (4)	C21—C22—C23—C24	1.7 (5)
N1—C4—C11—C16	148.0 (2)	C22—C23—C24—C25	1.7 (4)
C16—C11—C12—O1	−178.3 (3)	C22—C23—C24—C28	−178.6 (3)
C4—C11—C12—O1	5.8 (4)	C23—C24—C25—C26	−3.0 (5)
C16—C11—C12—C13	2.7 (4)	C28—C24—C25—C26	177.3 (3)
C4—C11—C12—C13	−173.2 (3)	C23—C24—C25—Cl2	177.3 (2)
O1—C12—C13—C14	−178.7 (3)	C28—C24—C25—Cl2	−2.4 (4)
C11—C12—C13—C14	0.3 (5)	C24—C25—C26—C21	0.8 (5)
C12—C13—C14—C15	−2.2 (4)	Cl2—C25—C26—C21	−179.5 (2)
C12—C13—C14—C17	179.1 (3)	C24—C25—C26—C27	−179.0 (3)
C13—C14—C15—C16	1.2 (4)	Cl2—C25—C26—C27	0.7 (4)
C17—C14—C15—C16	179.8 (3)	C22—C21—C26—C25	2.7 (4)
C13—C14—C15—Cl1	−178.4 (2)	C5—C21—C26—C25	−173.4 (3)
C17—C14—C15—Cl1	0.3 (4)	C22—C21—C26—C27	−177.5 (3)
C14—C15—C16—C11	1.8 (4)	C5—C21—C26—C27	6.4 (4)
Cl1—C15—C16—C11	−178.7 (2)		

### Hydrogen-bond geometry (Å, º)

**Table:**

*D*—H···*A*	*D*—H	H···*A*	*D*···*A*	*D*—H···*A*
O1—H1···N1	0.99 (5)	1.66 (5)	2.606 (3)	158 (4)
O2—H2···N2	0.86 (4)	1.83 (4)	2.619 (3)	152 (3)
C13—H13···O2^i^	0.95	2.59	3.464 (4)	152

Symmetry code: (i) −*x*+1, −*y*+1, *z*+1/2.

## References

[bb2] Groom, C. R. & Allen, F. H. (2014). *Angew. Chem. Int. Ed.* **53**, 662–671. 10.1002/anie.201306438 24382699

[bb3] Hutchins, R. O., Kopp, L. D. & Eliel, E. L. (1968). *J. Am. Chem. Soc.* **90**, 7174–7175.

[bb4] Kober, E., Nerkowski, T., Janas, Z. & Jerzykiewicz, L. B. (2012). *Dalton Trans.* **41**, 5188–5191. 10.1039/c2dt30153a 22437916

[bb5] Koll, A., Karpfen, A. & Wolschann, P. (2006). *J. Mol. Struct.* **790**, 55–64.

[bb6] Parsons, S., Flack, H. D. & Wagner, T. (2013). *Acta Cryst.* B**69**, 249–259. 10.1107/S2052519213010014 PMC3661305 23719469

[bb7] Rivera, A., Gallo, G. I., Gayón, M. E. & Joseph-Nathan, P. (1993). *Synth. Commun* **23**, 2921–2929.

[bb8] Rivera, A., Nerio, L. S. & Bolte, M. (2013). *Acta Cryst.* E**69**, o1166. 10.1107/S1600536813017157 PMC3770425 24046710

[bb9] Rivera, A., Nerio, L. S. & Bolte, M. (2014). *Acta Cryst.* E**70**, o243. 10.1107/S1600536814002128 PMC3998388 24764964

[bb10] Rivera, A., Nerio, L. S., Ríos-Motta, J., Fejfarová, K. & Dušek, M. (2012*a*). *Acta Cryst.* E**68**, o170–o171. 10.1107/S1600536811053748 PMC3254510 22259455

[bb11] Rivera, A., Nerio, L. S., Ríos-Motta, J., Kučeráková, M. & Dušek, M. (2012*b*). *Acta Cryst.* E**68**, o3043–o3044. 10.1107/S1600536812040329 PMC3470394 23125807

[bb12] Rivera, A., Nerio, L. S., Ríos-Motta, J., Kučeraková, M. & Dušek, M. (2012*c*). *Acta Cryst.* E**68**, o3172. 10.1107/S1600536812042808 PMC3515265 23284485

[bb13] Rivera, A. & Quevedo, R. (2004). *Tetrahedron Lett.* **45**, 8335–8338.

[bb14] Rivera, A. & Quevedo, R. (2013). *Tetrahedron Lett.* **54**, 1416–1420.

[bb15] Rivera, A., Quevedo, R., Navarro, M. A. & Maldonado, M. (2004). *Synth. Commun.* **34**, 2479–2485.

[bb21] Rivera, A., Quiroga, D., Ríos-Motta, J., Kučeraková, M. & Dušek, M. (2013). *Acta Cryst.* E**69**, o217. 10.1107/S1600536813000305 PMC3569752 23424498

[bb16] Rivera, A., Ríos-Motta, J., Quevedo, R. & Joseph-Nathan, P. (2005). Rev. Col. Quím. 34, 105–115.

[bb17] Rivera, A., Sadat-Bernal, J., Ríos-Motta, J., Pojarová, M. & Dušek, M. (2011). *Acta Cryst.* E**67**, o2581. 10.1107/S1600536811035677 PMC3201546 22065817

[bb18] Sheldrick, G. M. (2008). *Acta Cryst.* A**64**, 112–122. 10.1107/S0108767307043930 18156677

[bb19] Sheldrick, G. M. (2015). *Acta Cryst.* C**71**, 3–8.

[bb20] Stoe & Cie (2001). *X-AREA* and *X-RED32*. Stoe & Cie, Darmstadt, Germany.

